# Review of Catheter-Associated Urinary Tract Infections and *In Vitro* Urinary Tract Models

**DOI:** 10.1155/2018/2986742

**Published:** 2018-10-14

**Authors:** Yvonne J. Cortese, Victoria E. Wagner, Morgan Tierney, Declan Devine, Andrew Fogarty

**Affiliations:** ^1^Materials Research Institute, Athlone Institute of Technology, Athlone, Ireland; ^2^Bioscience Research Institute, Athlone Institute of Technology, Athlone, Ireland; ^3^Teleflex, Reading, PA, USA

## Abstract

Catheter-associated urinary tract infections (CAUTIs) are one of the most common nosocomial infections and can lead to numerous medical complications from the mild catheter encrustation and bladder stones to the severe septicaemia, endotoxic shock, and pyelonephritis. Catheters are one of the most commonly used medical devices in the world and can be characterised as either indwelling (ID) or intermittent catheters (IC). The primary challenges in the use of IDs are biofilm formation and encrustation. ICs are increasingly seen as a solution to the complications caused by IDs as ICs pose no risk of biofilm formation due to their short time in the body and a lower risk of bladder stone formation. Research on IDs has focused on the use of antimicrobial and antibiofilm compounds, while research on ICs has focused on preventing bacteria entering the urinary tract or coming into contact with the catheter. There is an urgent need for *in vitro* urinary tract models to facilitate faster research and development for CAUTI prevention. There are currently three urinary tract models that test IDs; however, there is only a single very limited model for testing ICs. There is currently no standardised urinary tract model to test the efficacies of ICs.

## 1. Introduction

The urinary system is one of the main routes through which the human body excretes liquid waste. The urinary tract is divided into two sections: the upper tract consists of the kidneys and ureters, where liquid wastes from the body are converted into urine and other products; and the lower tract includes the bladder and urethra, where urine is stored in the bladder before being expelled from the body through the urethra [1]. The outermost section of the urethra and the tissue surrounding the urethral opening are known as the urethral meatus [2].

When functioning normally, the lower urinary tract flushes out the urethra as the bladder empties, preventing the movement of bacteria up from the periurethral skin into the urethra and then into the bladder [3]. If bacteria manage to enter the bladder of a healthy individual, they will usually be expelled during micturition. However, if they remain, the bladder's internal surface is resistant to bacterial attachment as it is lined with urothelial cells that are covered in a glycosaminoglycan mucin that prevents bacteria adhering to the internal bladder surface [3]. In the event that a bacterium bypasses this first line of defences, the immune system should be able to eliminate the bacteria as long as the patient is healthy. If the immune system fails, a urinary tract infection (UTI) can occur and possibly lead to serious illness [3]. When problems arise in the lower urinary tract such as nerve damage or muscle atrophy leading to incontinence, or by prostate enlargement or urethral stricture resulting in urinary retention, the use of a urinary catheter becomes a necessity [1, 3].

## 2. Urinary Catheters

A urinary catheter is a long tube that can be constructed from any number of different polymers, with silicone being typically used, and latex rubber also common [4]. When required, the urinary catheter is inserted into the urethra as far as needed until the urine begins to flow. This is known as transurethral catheterisation. A catheter can also be inserted by a medical professional through the creation of an artificial track between the bladder and the abdominal wall, known as suprapubic catheterisation [3]. A urinary catheter can be a temporary or long-term solution depending on the patient's personal mobility and their prognosis.

**Figure 1:**
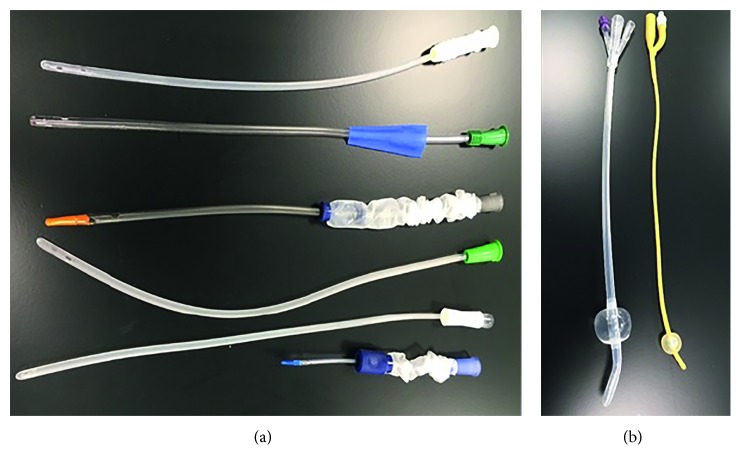
Various intermittent catheters (a): the upper five are male catheters, the lowest one is a female catheter. Two indwelling catheters with retention balloons inflated (b).

If a patient has the ability to take care of their own medical needs, temporary self-catheterisation can be the best option and performed easily [3]. For patients in which self-catheterisation is not an option, indwelling catheters become a necessity to maintain proper function of the urinary system. The most commonly used urinary catheter in the world is the Foley catheter that was invented by an American urologist named Frederic Foley [3]. The Foley catheter consists of a tube containing two channels; the larger channel allows the flow of urine from the bladder, and the smaller channel allows inflation of a balloon just below the tip of the catheter that, once inflated, holds the catheter in place until removed (Figure 1). Under optimal conditions, a urinary catheter can stay in place for up to approximately 12 weeks. However, this is often not the case as encrustation and bacterial infection can block the catheter or lead to medical complications [3].

## 3. Urinary Tract Infections

Catheters are one of the most commonly used medical devices. However, these devices are notoriously prone to infection. Infection is the largest concern with catheter use, whether long term or short term. Catheter-associated urinary tract infections, abbreviated to CAUTIs, are the most commonly faced hospital-acquired infections or nosocomial infections [5, 6]. CAUTIs can lead to numerous medical complications such as catheter encrustation, bladder stones, septicaemia, endotoxic shock, and pyelonephritis [5]. CAUTIs can be caused by yeasts or bacteria, including both Gram-positive and Gram-negative bacteria [4].

A study by Chatterjee et al. [7] sampled 150 catheters from patients with no history of UTIs and found that 130 of the catheters had pathogens present both on the catheter and in accompanying urine samples. The most common microorganisms found during the study by Chatterjee et al. [7] included “*Pseudomonas aeruginosa, Staphylococcus aureus, Staphylococcus epidermidis, Klebsiella pneumoniae, Proteus mirabilis, Proteus vulgaris, Escherichia coli, Citrobacter freundii, Providentia rettgeri,* and *Candida albicans*”. These bacteria can cause asymptomatic bacteriuria or UTIs which can be devastating to at-risk patients.

All catheter types and brands are vulnerable to CAUTIs, biofilm formation, or encrustation, and current methods to prevent these complications may just delay the process without treating the problem [8, 9]. Other approaches, such as prophylactic antibiotic courses, raise concerns about antimicrobial resistance and the evolution of numerous new resistant bacterial strains. This is especially relevant to resistance in the treatment of biofilm-linked infections [5, 10]. Additionally, the overuse of antimicrobials could disturb the balance in the bladders, naturally present microflora, and further contribute to pathogenesis [11].

The long-held idea that the bladder and urine itself are sterile is a misconception made by early bacteriologists in the 1800s [12]. This idea led a lot of doctors to believe that any UTI or CAUTI was from external contamination only. As the field of microbiology evolved, a better understanding of body's intricate microbiome has developed, e.g. *Corynebacterium* species in male and *Lactobacillus* species in female urinary tracts [12–15]. Today, it is understood that the prevalence of CAUTIs seems to be caused by a combination of both internal microflora and external introduced contamination [11]. Patients who practice intermittent catheterisation are most at risk from the microflora of the meatus being pushed up and into the bladder by catheter use, with *E.coli* being the main species responsible for CAUTIs in intermittent catheter users [16]. With indwelling catheters, the main concern is bacterial biofilms that lead to the creation of crystalline biofilms, with *P. mirabilis* infection being a lead concern to patients [10].

### 3.1. *Escherichia coli*

*E. coli* is a member of the *Enterobacteriaceae* family of bacteria that includes some of the best known pathogens that affect human health. *E. coli* is the most well-documented and studied bacterial species in the world, yet it still poses a consistent threat to human health, particularly in a medical setting [6]. Within human anatomy, *E. coli* is primarily found in the gastrointestinal tract (GIT). With the proximity of the urethra to the anus, especially in female patients, *E. coli* is a large contributor or initiator in the majority of CAUITs for intermittent catheter users [6].

Uropathogenic *E. coli* (UPEC) strains that are associated with UTIs are part of a subset of strains referred to as extraintestinal pathogenic *E. coli* strains, and they can cause sepsis and meningitis in addition to UTIs [6]. UPEC strains are amongst the most common isolates of nosocomial UTIs and the most common cause of UTIs in the general public. *E. coli* accounts for 70–90% of UTIs in the general public and 50% of all nosocomial UTIs [6]. *E. coli* is a motile bacterial species utilising flagellum-mediated motility to invade the urinary tract. When *E. coli* is introduced into the urinary tract on the surface of a catheter, it can move out into the bladder and can eventually move into the upper urinary tract, potentially causing kidney infections [6].

Once inside the body, UPEC strains exhibit a number of virulence factors that contribute to the formation and recurrence of UTIs and CAUTIs [6]. One such virulence factor is the expression of type 1 fimbriae, which are found in 80–100% of UPEC strains [6]. This adhesin allows UPEC strains and other uropathogens to adhere to the uroepithelial cells lining the urinary tract as well as the surface of a catheter [17]. The ability to adhere to the catheter allows for the establishment of a UPEC infection which can then support complex biofilm formation for UPEC and other strains [6]. Attachment to uroepithelial cells can lead to invasion and deterioration of the uroepithelial layer and perpetuation of CAUTIs after removal of the catheter [17]. UPEC strains are also capable of avoiding the host immune system through capsule and liposaccharide (LPS) production. The capsules produced by UPEC play an important role in UTIs and CAUTIs as the capsules aid in host immune avoidance, masking the bacterial cells with surface structural similarities to human cells and providing resistance to phagocytosis by immune cells [6, 18]. Capsules and LPS expression by UPEC strains has also been shown to aid in their resistance to complement-mediated lysis and endogenous antimicrobial peptides [6, 19].

### 3.2. *Proteus mirabilis*

*P. mirabilis* is a member of the *Enterobacteriaceae* family [6]. Species within the *Proteus* genus are widely distributed in the environment and opportunistic, having been linked to numerous nosocomial infections throughout the body [6]. *P. mirabilis* is normally not associated with UTIs in healthy persons with unobstructed urinary tracts [10]. *P. mirabilis* can, however, colonise the urinary tract of individuals who have structural or functional abnormalities. Catheterised patients are mostly at risk, as *P. mirabilis* can move along catheter surfaces from the outside, most likely due to existing colonisation of gastrointestinal tract [6].

*P. mirabilis* is the second most common bacterial strain isolated from CAUTIs in patients with long-term indwelling catheters, and of all Gram-negative bacteria, it tends to display the greatest propensity to bind the surface of catheters and urological devices in general [6]. The greater adherence abilities seen in *P. mirabilis* are due to its production of multiple adherence factors such as hemagglutinins and fimbriae, which allow *P. mirabilis* to attach to devices with or without the presence of a conditioning film [8]. The adherence ability of *P. mirabilis* plays a large role in CAUTIs in general and in particular, catheter encrustation/crystalline biofilm formation [6]. *P. mirabilis* also distinguishes itself from other uropathogens by its high motility and flagella-mediated swarming [20]. A study by Jones et al. [20] demonstrated under which conditions *P. mirabilis* swarm, which genes were involved, and visualised cells working together forming swarmer “cell rafts”. This work not only discussed the high motility of *P. mirabilis* but also demonstrated how the cells move together across a catheter surface by interweaving their flagella together into helical connections to move rapidly across a surface as one mass [20]. Jones et al. proposed that this movement not only contributes to the virulence of *P. mirabilis* but is also conducive to the movement of the bacterium from the skin, to the catheter, to the bladder [20]. *P. mirabilis* has also been observed to travel from the bladder to the kidneys and form kidney stones [10].

*P. mirabilis* produces urease, which hydrolyses urea, and is essential for crystalline biofilm formation in the urinary tract [21]. *P. mirabilis* has the highest production of urease out of all uropathogens, and the urease it produces is extremely reactive, hydrolysing urea faster than any other species, leading to rapid crystal formation that can encrust catheters and form bladder stones [21].

Many patients who experience recurrent CAUTIs and in particular recurrent catheter encrustation and blockage have been found to be carriers of *P. mirabilis* [9]. Sabbuba et al. [9] whilst investigating indwelling catheter patients in a nursing home found that *P. mirabilis* was continually isolated from the same patient even after catheter removal and antibiotic treatment. They found through genetic analysis that the same strain of *P. mirabilis* was found both within the crystalline biofilm of their encrusted catheters and in the patient's urine without the presence of a catheter [9]. These findings were later substantiated by another study by Sabbuba et al. [22] where they genotyped *P. mirabilis* strains isolated from bladder stones and compared them with *P. mirabilis* strains isolated from the same patient's encrusted catheters and found them to be identical; thus complications caused by *P. mirabilis* could be due to residual crystalline fragments in the urinary tract after catheter removal.

Another study by Mathur et al. [23] also isolated and genotyped *P. mirabilis* strains from patient urine and faecal samples. The patients tested were catheterised for at least 9 months prior to the study, and they found that out of the 18 patients included in the study, 10 tested positive for *P. mirabilis* in both urine and faecal samples [23]. Mathur et al. [23] also determined that strains of *P. mirabilis*, if isolated from both a faecal and urine sample of the same patient, were genetically identical, and as such it was proposed that faecal contamination from the patient themselves could be the cause of recurrent CAUTIs and catheter encrustation. Alternatively, this study also speculates that patients who do not experience catheter encrustation may not be faecal carriers of *P. mirabilis* [23].

## 4. Biofilms

The cellular structure of the bladder and the regular emptying of its contents usually prevent bacteria from multiplying to dangerous levels or adhering to the surrounding mucosa. When a foreign object like a catheter is introduced, bacterial contamination may occur [3]. Normally in the bladder, microorganisms are present in a planktonic state where they are freely suspended in the urine. In this state, they are unlikely to cause a UTI unless they are present in large numbers that may overwhelm the bladder's innate defences [4]. When an indwelling urinary catheter is in place, or any medical device within the body, microorganisms can attach to the medical device, forming vast colonies bound together and usually enclosed in a polymer matrix known as biofilms [4, 12].

A biofilm is defined as microorganisms bound to a surface of each other with the presence of an extracellular matrix composed of secreted products of the organisms and/or of components of the microorganisms themselves [24]. The cells within the biofilm may be irreversibly bound to the surface and to each other via secreted, adhesive substances [24]. The organisms contained within the biofilm usually demonstrate changes in gene expression differing from their planktonic state [25]. A biofilm can contain just one or multiple species, and the organisms involved can be Gram-negative or Gram-positive bacteria and yeasts [24]. The longer a urinary catheter is in place, the more likely it is for a biofilm to form on its surface and cause a CAUTI. Patients who are catheterised short term (≤7 days) experience biofilm formation 10–50% of the time; however, practically all patients who are catheterised long term (>28 days) are found to present with biofilm formation [4].

Being a part of a biofilm is highly advantageous to a microorganism, as the group together is much more resilient and resistant than any singular planktonic organism [4, 17]. The advantages for an organism being within a biofilm community include antimicrobial resistance, protection from physical forces, and safety from phagocytosis by immune cells [25]. The ability of biofilms to resist antimicrobial agents is particularly worrying, as mechanisms of resistance, such as genes encoding for antimicrobial resistance, can be transferred throughout the community and even further afield as microorganisms leave the biofilm to spread and multiply [24]. Within a biofilm, cell-to-cell communication can occur in a process known as quorum sensing to choreograph changes in the gene expressing across the community [6]. A biofilm can also influence and change aspects of the surrounding environment and is of particular interest when examining the factors that lead to encrustation and blocking of urinary catheters [26].

## 5. Crystalline Biofilms

Urinary catheter encrustation is an ongoing problem with no simple solution. Catheter encrustation can cause numerous unfavourable outcomes for patients beyond the previously mentioned health risks biofilms alone present. Encrusted catheters become blocked, leading to urine retention that is not just painful for the patient but also constitutes a medical emergency [3]. Once blocked by encrustation, the catheter must be removed to avoid damaging the bladder, ureters, and kidneys; if the pressure builds to a high enough level in the bladder, ureteric reflux can occur where urine is forced backwards up into the ureters and into the kidneys [3]. In some patients, catheter crystallisation can be so extreme that removal of the catheter can require emergency surgery [27].

**Figure 2:**
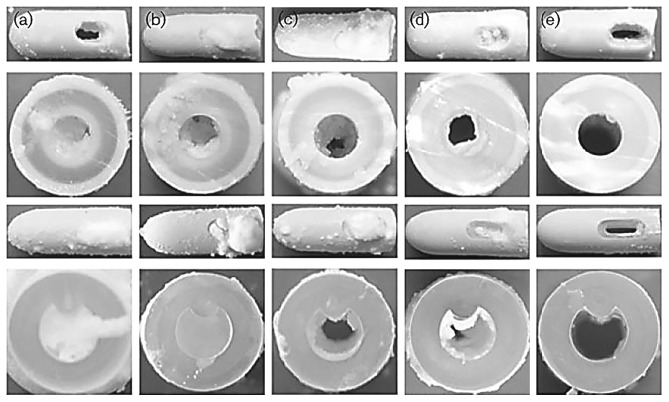
Examples of catheters encrusted by crystalline biofilms created by various bacteria: (a) *Proteus mirabilis,* (b) *Proteus vulgaris,* (c) *Providentia rettgeri,* (d) *Morganella morganii,* and (e) *Staphylococcus aureus*. The top two rows are silver/latex catheters, and the bottom two rows are nitrofurazone/silicone catheters [26].

Encrustation can occur due to metabolic dysfunction, but generally urinary catheter encrustation occurs due to bacterial influence, particularly urease-producing bacteria that form crystalline biofilms (Figure 2) [14]. Although various bacterial and yeast strains can and do lead to UTIs or CAUTIs, when it comes to urinary crystalline catheter encrustation, the urease-producing species *Proteus mirabilis, Proteus vulgaris,* and *Providentia rettgeri* are of interest in most studies [5, 18, 19, 20]. Of these three bacteria, *P. mirabilis* is isolated most frequently from patients and produces the most urease, an enzyme that hydrolyses urea, breaking it down into ammonia and carbonate ions [27]. Urease-producing bacteria use the produced ammonia as a source of nitrogen and carbon to support further colony growth [6]. Increasing ammonia levels lead to an increase in the overall pH of the urine in the bladder, and the bacterially produced alkaline environment causes calcium and magnesium to come out of solution and precipitate into crystals [28]. The crystallised mineral forms of magnesium and calcium are known as struvite, which is magnesium ammonium phosphate, and apatite, a poorly crystallised form of hydroxylated calcium phosphate [27]. This process of catheter encrustation via crystallisation is directly connected to the formation of biofilms and the products produced by the organisms within [28].

As with the advantages for the bacteria to form into a biofilm, there are also specific advantages to forming crystallised biofilms. CAUTIs can often persist in patients when a catheter is removed, and several studies believe this could be due to the crystalline biofilm formation [26, 27]. As the previously encrusted catheter is removed, crystals can break off, still containing the bacterium that they formed upon [29]. These crystal fragments act as a nuclei on which newly forming minerals can grow and ultimately form bladder stones [29]. These bladder stones can store pathogens, reinfecting the bladder and allowing the biofilm crystallisation of a new catheter, thus perpetuating the cycle [29]. Morris et al. [28] and more recently Barros et al. [30] describe the development of a crystallised biofilm on the surface of urinary devices as follows:

1. The urinary tract is infected by a urease-producing bacterial species
2. The surface of the catheter is prepared for bacteria adhesion by the production of an organic conditioning film by the deposition of urine components, ions, and minerals
3. The urease-producing bacteria adhere to the conditioning film
4. The biofilm community begins to form as they excrete an exopolysaccharide matrix
5. As bacterial numbers rise in the biofilm, so does the release of urease that goes on to hydrolyse urea into ammonia, increasing the pH of both the urine and the biofilm
6. Calcium and magnesium ions are attracted to the biofilm's gel matrix
7. The calcium and magnesium phosphate crystallise, forming struvite and apatite crystals on the device's surface bound with the biofilm

## 6. Noncrystallised Biofilms

Some urease-producing bacterial species do not form crystallised biofilms as their urease production levels are too low. These include *Pseudomonas aeruginosa, Staphylococcus aureus, Klebsiella pneumoniae, Escherichia coli, Morganella morganii,* and *Providencia stuarii,* to name a few [13]. While these microbes do have the ability to form a biofilm, it will not be crystalline in structure without help from other species as their lower urease output, although able to hydrolyse urea into ammonia, is not high enough to raise the urine to a pH of >8.0, which is needed for apatite and struvite to form [26].

Interestingly, *Klebsiella pneumoniae* and *Pseudomonas aeruginosa,* although they cannot produce crystals, can still block catheters and cause the same problems associated with reduced or halted bladder drainage [27]. Broomfield et al. [26] investigated different approaches to controlling crystalline biofilms on catheters, and during their testing, they observed that both *Klebsiella pneumoniae* and *Pseudomonas aeruginosa,* while not able to produce a crystalline biofilm, produced large amounts of a mucoid material that did not block the catheter but did greatly reduce urine flow.

## 7. Control and Prevention of Catheter-Associated Urinary Tract Infections

Due to the ongoing problems caused by crystalline biofilm encrustation with indwelling catheters and CAUTIs in general with intermittent catheters, numerous studies have been carried out proposing novel solutions and many manufacturers have come out with new products, all with varying levels of success [7, 19, 23, 26–40].

### 7.1. Indwelling Catheters

IDs are primarily at risk of CAUTIs due to biofilm formation. Copious amounts of research to date has been carried out to develop and test novel indwelling catheters to prevent CAUTIs, more specifically bacterial adherence, biofilm formation, and catheter encrustation [6, 9, 10, 18, 22–24, 31]. These are generally characterised by the antimicrobial agent, material, design, or practice involved.

#### 7.1.1. Ceftazidime, Ceftriaxone, Cisplatin, Heparin, and Nitrofurazone

Over the decades, many studies and manufacturers have tried to produce a urinary catheter that resists biofilm formation by impregnation or coating the catheter in an antibiotic, antimicrobial, or bactericidal compound. A study by Ghanwate et al. [32] investigated the efficacy of four antimicrobial agents effective against the urinary pathogen *P. aeruginosa* to determine if they could prevent or remove biofilm formations on coated catheters. The antibiotics ceftazidime and ceftriaxone were found to postpone biofilm formation for 14 days and 8 days, respectively [32]. Cisplatin is a medication used commonly in chemotherapy treatment but can also be effectively used as an antimicrobial. In the study by Ghanwate et al. [32], cisplatin prevented biofilm growth for 8 days. Finally, Ghanwate et al. [32] looked at the antimicrobial ability of heparin, normally used as an anticoagulant, and the antibiofilm enzyme DNase. They found that heparin prevented biofilm formation for only 6 days and DNase only for 5 days, making them the least effective long term. The compounds identified by the work of Ghanwate et al. [32] could be useful for short-term or intermittent catheterisation but are not effective for long-term catheterisation or >28days. Although several studies suggest that the antimicrobial nitrofurazone might be a suitable antimicrobial agent to coat catheters [35], its effects against *P. mirabilis* are negligible at best, and it does not prevent crystalline biofilm formation [13].

#### 7.1.2. Silver

Another antimicrobial that has been used in catheters is silver, either impregnated into the catheter's materials itself or as part of a hydrogel coating on the catheters surfaces [4, 18]. The antimicrobial effects of silver have long been utilised in medical dressings for burns and pressure ulcers as well as consumer goods [33]. Silver exhibits broad-spectrum antimicrobial activity that is effective against both anaerobic and aerobic bacteria as well as both Gram-positive and Gram-negative bacteria [33]. Silver's mechanism of action involves the release of ions that cause oxidative damage to a bacterium's cellular DNA and disruption of the cell's membrane [36]. Silver is medically important as it can be effective against antibiotic-resistant strains like methicillin-resistant *Staphylococcus aureus* (MRSA); however, some bacteria are now showing resistance to silver, including *Proteus mirabilis, Enterobacter cloacae, Citrobacter freundii, and Klebsiella pneumoniae,* with *Proteus mirabilis'* resistance to silver of particular interest to preventing CAUTIs [33]. Some studies have found that silver can be effective in controlling bacterial levels during short-term catheterisation; however, the silver was found to only delay the onset of bacteriuria and has not been proven effective in prevention of CAUTIs [41, 42].

When discussing catheter research, different forms of silver coatings have been trialled. Silver oxide, silver alloys, and silver nanoparticles (AgNPs) have all been assessed to determine their efficacy in preventing bacteriuria, CAUTIs, and biofilm formation [41, 43–47]. Silver oxide catheters are no longer in the market, as any evidence they were effective in prevention of CAUTIs was deemed statistically insignificant [41]. A more promising silver catheter design contains a silver alloy where the silver has been stabilised by other metals such as gold and palladium, which allows for the slow release of silver ions [48]. A notable catheter with a silver alloy coating is the Bardex IC catheter which is coated in a silver alloy embedded in a hydrogel and palladium layer on both the internal and external surface [49]. Some studies in the past have demonstrated that silver alloy catheters postpone development and reduce instances of asymptomatic bacteriuria, while this may make these catheters seem like they could prevent CAUTIs, that has been categorically dismissed by several large-scale reviews [41, 42, 46, 49, 50]. More recently, a couple of studies have coated catheters in AgNPs to assess the antibiofilm or antifouling ability of the silver nanoparticles [43, 44]. In their laboratory study, Wang et al. [44] coated catheter segments with layers of AgNPs immobilised on polydopamine and an outer “antifouling layer” of Poly(sulfobetaine methacrylate-co-acrylamide). Their belief was that the outermost layer would prevent catheter encrustation and the silver would prevent CAUTIs over time. Wang et al. [44] found that their AgNPs coating reduced overall bacterial concentrations when compared with a control by two orders of magnitude and that their coating resisted encrustations for up to 45 days depending on how many AgNP layers were present. Another laboratory study by Thomas et al. [43] also coated catheter segments in AgNPs and found the coating inhibited the adhesion of coagulase negative staphylococci to the catheter with an 80–90% reduction in biofilm formation dependant on the test species.

#### 7.1.3. pH Control and Citrate

As discussed previously, many uropathogenic bacteria produce urease which hydrolyses urea, increasing the pH of the urine and ultimately leading to precipitation of calcium and magnesium in the urine and encrustation of catheters [51]. The pH at which calcium and magnesium precipitate in the urine is classified as the nucleation pH or pH_n_ [52]. Mathur et al. [52] determined that the pH_n_ varies in each individual and can account for why some patients block catheters very quickly while others do not. They found that the mean pH_n_ of participant's urine ranged from 6.67 to 8.96. In addition to the fact that the pH_n_ of a patient's urine could vary from week to week suggested that the manipulation of pH_n_ could be a strategy for controlling encrustation [52]. Mathur et al. [53] went on to undertake another study where they assessed if lowering calcium and magnesium concentrations in the urine would affect the pH_n_ and thus reduce/eliminate catheter encrustation. They found that by increasing a patient's fluid intake, there would be a decrease in magnesium and calcium concentration in the urine and a resulting increase in the patient's pH_n_ [53]. They also found calcium concentration to have a larger impact on pH_n_ than magnesium [53].

A study by Suller et al. [51] also investigated the link between pH_n_ and catheter encrustation with the aim to control a patient's pH_n_ with citrate. Citrate acts as a chelating agent for divalent metal ions, and as such it can keep calcium and magnesium in solution [27, 54]. Suller et al. [51] found that the intake of citrate can increase a patient's pH_n_, decreasing the likely hood of their catheter blocking. Intake of fresh orange juice, daily, would cover the recommendations of both which Suller et al. [51] found could raise a urine pH_n_ from 7.24 to 8.2 reducing the risk of catheter encrustation. Another later study by Khan et al. [54] found similar results when assessing the impact on patient pH_n_ when administered citrate in the forms of lemon juice and potassium citrate. They came to the same conclusion as that from the work carried out by Suller et al. [51] that increased citrate and fluid intake is effective in modulating a patient's pH_n_ and could be an inexpensive, simple, and effective way to deal, reduce, and control catheter encrustation [54].

A study by Broomfield et al. [26] specifically looked at the urease production levels of uropathogens in relation to catheter blockage time. They found that the thirteen bacterial species tested fell into three distinct groups based on levels of urease production, which also correlated to the largest changes in urine pH recorded [26]. The highest urease-producing species were *Proteus mirabilis, Proteus vulgaris*, *and Providencia rettgeri* and all blocked catheters quickly and completely. The use of citrate and increased fluid intake to increase pH_n_ and reduce encrustation is part of the advice given to catheterised patients for over 20 years [13]. Although this solution successfully reduces catheter encrustation *in vitro* and *in vivo*, it only addresses the crystallisation but not the bacterial infection itself.

#### 7.1.4. Triclosan

Triclosan is biocide with antimicrobial capabilities and can act as both an antibiotic and an antimycotic [37]. Triclosan, like the other antimicrobials discussed, has also been used in several studies to control catheter encrustation and biofilm formation [2, 5, 26, 55, 56]. It has been well established that *P. mirabilis* is highly susceptible to triclosan, and it can be used as an alternative to antibiotic treatments [55]. One method of triclosan administration comprises filling the balloon of a Foley catheter with a triclosan/polyethylene glycol solution instead of water, allowing the triclosan solution to permeate out of the catheter balloon slowly [56]. With *P. mirabilis'* susceptibility to triclosan so well documented, a study by Broomfield et al. [26] aimed to also test triclosan's efficacy against the other two main urease-producing strains, *P. vulgaris,* and *P. rettgeri*. Their study found that *P. vulgaris,* like *P. mirabilis,* is fully sensitive to triclosan, and catheter blockage was prevented, while *P. rettgeri* was resistant to the triclosan levels achievable in the bladder, and catheter blockage occurred [26]. Even with these positive results, triclosan is not the final solution in encrustation defence, since as with all antimicrobial use, microbial resistance is a never-ending race that triclosan may already be losing [37]. Triclosan's mechanism of antimicrobial action is also concerning as it targets the enzyme enoyl reductase, which is required by most bacteria for fatty acid biosynthesis. Numerous antibiotics also work by targeting enoyl reductase, and if microbes develop resistance to triclosan, they may develop cross resistance to a variety of antibiotics [37].

#### 7.1.5. Furanones

Quorum-sensing communication contributes to the development of biofilms, and some studies have looked towards furanones as a solution to disrupting a bacterium's ability to communicate to prevent or inhibit biofilm formation [25]. Furanones are naturally occurring compounds that are secreted by the red algae species *Delisea pulchra* that possess the ability to disrupt cell-to-cell signalling, thereby inhibiting biofilm growth in several microbe species. Some studies have shown that *E. coli* exposed to furanones demonstrated a clear reduction in biofilm thickness, and when *P. aeruginosa* was exposed to furanones, quorum sensing-controlled gene expression was reduced in a manner that impacted *P. aeruginosa*'s biofilm architecture and total biomass [57, 58]. Furanones also inhibited the biofilm growth of *S. epidermidis, B. subtilis,* and *E. coli;* however, more research studies are needed to determine if the cause of inhibition is in fact quorum-sensing disruption [57, 58]. Unfortunately, the potential toxicity of furanones has resulted in their limited clinical use [6, 10].

#### 7.1.6. Polymers and Biomaterials

Throughout the modern history of catheter use, numerous materials have been tested to assess their ability to prevent UTIs or CAUTIs, including polyurethane, hydrogels, silicone, latex, and composite biomaterials [4]. No material alone has presented a universal solution; however, new innovations in material science and, in particular, polymer science may hold the key. One such biomaterial catheter was studied by Vapnek et al. [59]; LoFric^®^ catheters covered in a hydrophilic coating were found to have a decreased incidence of UTIs or CAUTIs in clinical settings. A study by Stickler [60] investigated another hydrophilic polymer catheter that was coated in phosphorycholine, which is the main polar head group found in erythrocytes. This phosphorycholine-coated catheter was not only biocompatible with the body but it was also stable. Although it has been shown to inhibit bacterial colonisation on medical devices like contact lenses; in urological tests, it did not inhibit the growth or formation of crystalline biofilms by *P. mirabilis* [13, 57, 58, 61].

#### 7.1.7. Probiotics

The use of nonpathogenic bacterial species as probiotics to displace pathogenic bacteria has had some successes in preventing UTIs. One study found that 21 patients who had been inoculated with *E. coli* 83972 experienced no bacteriuria when compared to the previous year where an average of 3.1 patients suffered a UTI [62]. Another study by Darouiche et al. [63] also discusses the success of nonpathogenic *E. coli* strains to prevent UTIs in patients with neurogenic bladders caused by spinal cord injuries. They found that direct insemination of *E. coli* into the bladder was safe and did not produce symptoms of a UTI, but it did lower the overall instances of UTIs when compared with the patients' history [63]. This probiotic approach could be an effective tactic in the future.

#### 7.1.8. Alternative Designs

The disruption of CAUTI biofilms by mechanical catheter-pathogen displacement has been investigated as a potential CAUTI solution. Chakravarti et al. [64] investigated the use of an electrical current to aid in the dispersion of silver ions from a silver-impregnated catheter. The method works by passing an electric current through silver electrodes imbedded in the catheter, which generate silver ions in the bladder [64]. This method was effective in inhibiting the biofilm growth on the catheter; however, the effect was temporary as the electrical current caused the silver electrode to disintegrate after 150 hours, making this method only suitable for short-term catheterisation [64].

An alternative mechanical approach was reported in a study by Hazan et al. [65], where an elastic wave-generating actuator was attached to a Foley catheter before insertion into the urethra of a male rabbit. The actuator produced low-energy acoustic waves along the surface of the catheter, which was found to prevent biofilm production for up to 9 days in contrast to the control animals' catheters, which had biofilm formation by the second day [65].

Another study by Levering et al. [66] developed a novel ID design that aimed to disrupt and dislodge biofilms from the catheter's inner drainage lumen. Their catheter was designed to have inflation lumens which run the length of the catheter in between the urine drainage lumen and the outer catheter wall [66]. When inflated, they found that the pressure exerted by the inflation lumens was sufficient to dislodge crystalline biofilms from the inner luminal surface, breaking them apart, so the remnants could then be flushed out by the flow of the patient's urine [66].

Sun et al. [67] developed a new ID, but unlike the last study discussed, they focused on the catheter's outer surface structure. They refer to their novel catheter design as “trefoil-shaped” with three longitudinal grooves that run the length of the catheter [67]. According to Sun et al., this trefoil shape would allow bladder drainage like any other catheter, but it would cause less tissue damage as a lower surface area is in contact with the urethral mucosa, which in turn preserves normal tissue function and lowers risks of infection [67]. The trefoil design also enables normal secretions from the urethra that are not possible with the complete surface area contact of traditional IDs [67].

Another alternative design is the “Poiesis Duette,” which is an ID with two balloons, one for retention like other IDs and one above the drainage eyelet to protect the bladder urothelium [68]. Maintaining the integrity of the bladder, urothelium is integral to prevention of CAUTIs as once compromised, it can allow invasive pathogens to colonise the urinary tract and potentially cause irreversible damage [6, 66–68].

### 7.2. Intermittent Catheters

ICs are often seen as a solution to CAUTIs caused by indwelling catheters, as intermittent catheters pose no risk of biofilm formation due to their short time in the body and have a lower risk of bladder stone formation [69]. Although there is a lower incidence of CAUTIs with intermittent catheter users, there is still a high risk of CAUTI with infection rates as high as 60%. Recurrent CAUTIs are quite common with the inappropriate use of antibiotics [69]. While indwelling catheter research is extensive with a diverse array of possible solutions to CAUTIs, the research in respect to intermittent catheters is lacking in comparison. Indwelling catheter research and development as previously discussed is primarily focused on the use of antimicrobial and antibiofilm compounds to prevent catheter colonisation, or at least extend the time before colonisation occurs. Intermittent catheter research and development traditionally and currently is focused on preventing bacteria entering the urinary tract or coming into contact with the catheter, rather than treatment of CAUTIs [69].

#### 7.2.1. No-Touch Closed Systems

Some intermittent catheters are enclosed in closed systems consisting of what is known as a “no-touch” sleeve that allows insertion of the catheter by the patient or medical professional without needing to ever touch the catheter itself, therefore maintaining sterility of the catheter [69]. A study by Hudson and Murahata [38] compared hydrophilic-coated intermittent catheters to intermittent catheters that had physical barriers. The catheters that had a physical barrier contained a self-contained lubricant and a “no-touch” sleeve that covered the entirety of the catheter so that bacteria could not be transferred from the hands to the device. Their study demonstrated that hydrophilic coatings reduced the number of bacteria entering the urethra compared with the bacterial levels inoculated on the technician's hands. However, they found a significant reduction of pathogen numbers with the no-touch catheters having on average <5.4 colony-forming units, a huge decrease when compared with the hydrophilic-coated catheters at an average of 3.3 × 10^2^ colony-forming units [38]. A hybrid catheter that contained a hydrogel coating and a small plastic sleeve that the patient could use to avoid touching the catheter performed better than those with the hydrogel alone, but still were not as effective as the “no-touch” catheters, with an average of 2.5 × 10^1^ colony-forming units (CFUs). The results Hudson and Murahata [38] obtained demonstrate the effectiveness of complete physical barriers at dramatically displacing pathogens.

#### 7.2.2. Insertion Tip

**Figure 3:**
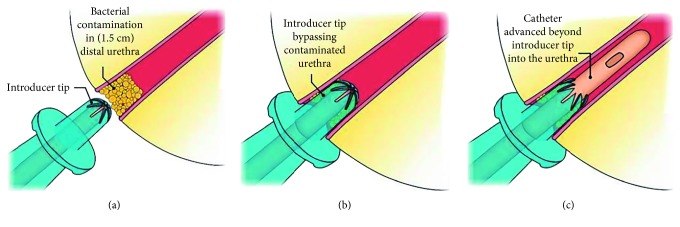
Insertion tip found on some intermittent catheters: (a) large bacterial numbers at urethral meatus, (b) insertion tip bypasses meatal bacteria, and (c) catheter passes through the insertion tip up the urethra towards the bladder [69].

Another study reported in a monograph by Holland and Fish [39] compared two catheters produced by the same manufacturer. One catheter was the Coloplast Self-Cath^®^, a generic PVC catheter with no added lubricants or sleeve. The second one was the Bard^®^ Touchless^®^ Plus intermittent catheter that is enclosed in a no-touch sleeve and includes an insertion tip [39]. Insertion tips were developed first in 1982 and are now often used as the tip allows the catheter to avoid the urethral meatus during insertion [69]. The urinary meatus includes not only the area directly around the urethral opening but also the first 1.5 cm inside the urethra, where high concentrations of bacteria are found (Figure 3) [69]. The study by Holland and Fish [39] assessed both the effectiveness of the insertion tip and that of the no-touch system to prevent pathogen transfer from the meatus and from the hands, much like the study by Hudson and Murahata [38]. From their work, Holland and Fish [39] determined that the no-touch catheter with the insertion tip performed significantly better in the insertion tip test with only 1.8 CFUs transferred to the catheter after insertion through an agar plate inoculated at 1 × 10^7^ CFUs, while the generic catheter yielded an average of 22 CFUs. In regards to the contaminated handling test, which was performed in a similar fashion to that of Hudson and Murahata [38], the no-touch catheter outperformed the generic catheter with 0 CFUs and 144 CFUs, recovered from the catheter after handling, respectively [39].

#### 7.2.3. Hydrophilic Coatings

Hydrophilic-coated intermittent catheters have been around since the early 1990s and have since become a standard in the industry [31, 32]. Several studies have demonstrated that hydrophilic-coated intermittent catheters do reduce incidences of CAUTIs or at least delay the onset. One such study by De Ridder et al. [70] found that only 64% of patients using a hydrophilic-coated intermittent catheter experienced one or more CAUTIs in a year compared with 82% of patients using uncoated PVC catheters. Similar results were reported by Cardenas et al. [71], which reported a 22% reduction of symptomatic CAUTIs by using hydrophilic-coated catheters. Finally, a meta-analysis by Li et al. [72] reported a correlation between lower incidences of CAUTI when using hydrophilic catheters from their analysis of five clinical studies. The lower incidences of CAUTIs found when using hydrophilic catheters could be a product of high patient satisfaction, as the hydrophilic coating can reduce pain, making the process easier and increasing a patient's quality of life that results in adherence to clean intermittent catheterisation [32, 36, 73, 74]. The lower incidence of CAUTIs may also be due to the hydrophilic-coated catheters not requiring additional external lubrication, and so there is no additional need to touch the catheter before insertion [69].

#### 7.2.4. Antimicrobial Coatings

Antimicrobial-coated catheters are primarily only produced for indwelling catheters, and there are a few available for intermittent catheters as the market shifts towards physical barriers and no-touch engineering [69]. There was at one point a hydrophilic intermittent catheter coated in the antimicrobial nitrofurazone, but this was later taken off the market [69]. Silver has been incorporated into or used to coat catheters for many years due to its broad-spectrum antimicrobial abilities [47]. Regev-Shoshani et al. [75] found silver-coated indwelling catheters ineffective against *E. coli.* The study by Ogilvie et al. [40] however contradicts the majority of research illustrating silver's ability to not only prevent *E. coli*'s ability to adhere to an indwelling catheter but also prevent biofilm formation. With all the historic and recent studies, there is a significant gap in the information where antimicrobial intermittent catheters are concerned [12, 31, 37].

#### 7.2.5. Reuse of Intermittent Catheters

Although the studies discussed show the advantage of enclosed and disposable catheters, due to financial issues, health insurers, environmental concerns, or just personal choice, some people use reusable intermittent catheters [69]. Although single-use sterile catheters are becoming the norm in many developed countries, they are not readily available in most developing and underdeveloped countries depending on the health-care system [76]. While some studies report higher incidences of CAUTIs with reusable catheters, others report no statistical difference in sterile single-use and reusable catheters, though this may be more so due to lack of evidence in the area with much catheter reuse under reported [31, 35, 40]. The primary cause for concern with catheter reuse is that there is no standard procedure for the patient to clean, sanitize, or resterilise the catheter after use [69]. Numerous techniques for sterilising reusable catheters are thought to be effective such as microwaving, boiling, and soaking in antiseptics such as alcohol, hydrogen peroxide, and bleach [41]. However, there has been little research published to prove the efficacy of any available technique [31, 77]. Sherbondy et al. [78] investigated the variations in technique of patients who practiced clean intermittent catheterisation with reusable catheters and utilised microwave sterilisation. They noted a large variation in both time and microwave wattage levels between patients demonstrating the lack of a standard methodology or instruction given to patients. A study by Bogaert et al. [79] performed several experiments to determine not only the antimicrobial efficacy of microwave heating and alcohol immersion sterilisation but also investigated the effects on the structure of the catheter, as often patients will reuse catheters that were never meant for reuse. Their work determined that microwave heating was adequate at eliminating *E. coli* but was not effective for *P. aeruginosa* or *S. aureus*, while having minimum effect on the catheter's physical properties [79]. In respect to alcohol immersion, they found that immersion in 70% alcohol was effective against all bacterial strains used and would be an appropriate sterilisation technique; however, immersion times should be kept short as the alcohol was found to create significant changes to the catheter's physical qualities [79].

### 7.3. Prophylaxis

#### 7.3.1. Prophylactic Antibiotic Use

Prophylactic systematic antibiotics use is common in both indwelling and intermittent catheter users [41]. Though more common in indwelling catheter patients with highly resistant or recurrent biofilms, intermittent catheter users are often given prophylactic treatments without any symptoms of UTI [31, 77]. Prophylactic antibiotics can be useful in some cases, and some studies have shown that their use can reduce CAUTI cases or offset the time before a problematic infection sets in [41]. That said, prophylactic antibiotic use is no longer recommended as it can contribute to the development of resistant microbial strains, as well as adverse outcomes for the patient such as recurrent and resistant infections as treatment often does not completely eradicate the offending organisms [31, 80].

#### 7.3.2. Prophylactic Antimicrobial Use

Prophylactic use of nonantibiotic antimicrobials is common for catheter users as well as community UTI sufferers. Cranberry products have long been a home remedy for the treatment and prevention of UTIs, and many catheterised patients utilise them in the hopes of preventing CAUTIs [41]. Proanthocyanidins are the active ingredient in cranberries that acts as an antiadherent for bacteria within the urinary tract due to their tannin molecules containing irregular A-type linkages, which prevents adhesion of bacteria to the inner walls of the bladder [69]. The majority of the studies on the effectiveness of cranberry, and in particular proanthocyanidins, is inconclusive or insufficient at best, and is well covered by both Goetz et al. [69] and Hooton et al. [41].

Another prophylactic treatment used is d-mannose, a sugar that plays an important role in human metabolism, and like proanthocyanidins, it can prevent bacterial adhesion to uroepithelial cells by binding to the type 1 pili of enteric bacteria [80]. A study by Kranjčec et al. [80] found a correlation with d-mannose use and a reduction in recurrent UTIs in their trial. A more recent study found that d-mannose in combination with other plant extracts, including arbutin, forskolin, berberine, and birch, was effective in reducing recurrent UTIs and could be an alternative to prophylactic antibiotic use or in fighting resistant bacterial infections [81].

Methenamine salts, either in the form of methenamine mandelate or methenamine hippurate, are another group of antimicrobials used in the past for control or prevention of UTIs and CAUTIs [41]. The antimicrobial mechanism of methenamine is due to its hydrolysis in the body to form ammonia and formaldehyde [41]. The formaldehyde formed is bactericidal and broad spectrum. It is also unlikely to lead to the development of resistant strains, as formaldehyde is an antiseptic rather than an antibiotic [77]. Many studies have looked at the efficacy of prophylaxis with methenamine, and the results are inconclusive, with some studies finding that methenamine can lower the risk of or prolong time before a UTI/CAUTI develops, while other studies found it no more effective than placebos [41]. Due to these inconclusive results, methenamine use was discouraged in the past; however, relatively recently, there has been renewed interest in nonantibiotic treatments due to increasing antibiotic resistance and stagnating development in discovery of new antibiotics [77].

## 8. Microbial *In Vitro* Catheter Testing and Urinary Tract *In Vitro* Models

*In vitro* testing is a key component of catheter research and development as it allows for potentially high throughput testing and initial screening for proving novel concepts, while avoiding the ethical issues and higher costs associated with *in vivo* testing [16, 69]. While current *in vitro* testing is not capable of truly representing the clinical realities of catheter use, it can provide valuable information in the early stages of novel catheter development [82]. *In vitro* testing in the area of urological devices and more specifically CATUIs and catheters includes a wide variety of general microbiological assays that are not necessarily specific to the field. Currently, there exist four *in vitro* models with the aim of more closely mimicking the anatomy of the urinary tract.

### 8.1. General Microbiology *In Vitro* Testing

General microbiology *in vitro* assays are often used or adapted to test novel urological medical devices and to detect infections in patients [12, 16, 41–43, 69].

#### 8.1.1. Microtitre Plate Biofilm Formation

The microtitre plate biofilm formation assay is a simple technique to form biofilms within the wells of a microtitre plate [16, 69]. The assay consists of a microtitre plate of 96, 48, 24, 12, or 6 wells, and bacteria are grown within the wells and bind either to the walls/base of the wells, or are introduced to items such as a catheter segments contained in plates with 24 wells or less [31]. The assay is high throughput whilst also relatively cheap and straightforward when compared to other biofilm models; however, the assay can be vulnerable to large variations from well to well [16, 69]. A macroversion of this assay also exists where test tubes are used in place of microtitre plates [24].

#### 8.1.2. Time to Kill Attached Bacteria Assay (tK100)

The tK100 assay establishes the time it takes for an antimicrobial to kill 100% of bacteria attached to a urological device [83, 84]. The assay protocol starts with segments of the test device, either uncoated or coated with a protein conditioning film, being exposed to a bacterial inoculum and allowing the bacteria to adhere to the device for a set period of time [41, 43]. Once bacteria are attached to the surface, the segments are washed to remove planktonic bacteria, and then the segments are placed in growth media and incubated for a set period of time, usually over several days [41–43]. Replicates are removed at set time intervals over the extended incubation time, then sonicated, and the remaining live bacteria enumerated. These bacterial counts are then plotted over time to establish the time intervals to reach 100% bacterial death [41–43].

#### 8.1.3. Serial Plate Transfer Test

The serial plate transfer test relies on producing zones of inhibition on a series of plates over time to establish both the duration of an antimicrobial's activity and the time taken to develop resistance [85]. The method is used in several studies, and the protocol starts with a segment of a device that is either imbedded or coated with an antimicrobial, and the segment is placed on an inoculated agar plate and incubated overnight [41, 42]. After incubation, the zone of inhibition, or area around the segment where no bacteria grew, is measured and recorded before the segment is transferred to a new inoculated plate and incubated again [41, 42]. This process is repeated with new plates each day, while making sure the same side of the segment is in contact with the agar each time it is transferred, until a zone of bacterial inhibition no longer forms [41, 42].

### 8.2. *In Vitro* Anatomical Models

Essentially, four models of the urinary tract or sections of the urinary tract and their adaptations have been developed in an attempt to more closely represent the anatomy and conditions *in situ* [28, 44–46]. These models are the bladder model by Stickler et al. [86], the urinary tract model by Gaonkar et al. [2], the CAUTI model by Rosenblatt et al. [87], and the meatus model by Holland and Fish [39].

#### 8.2.1. *In Vitro* Bladder Model

**Figure 4:**
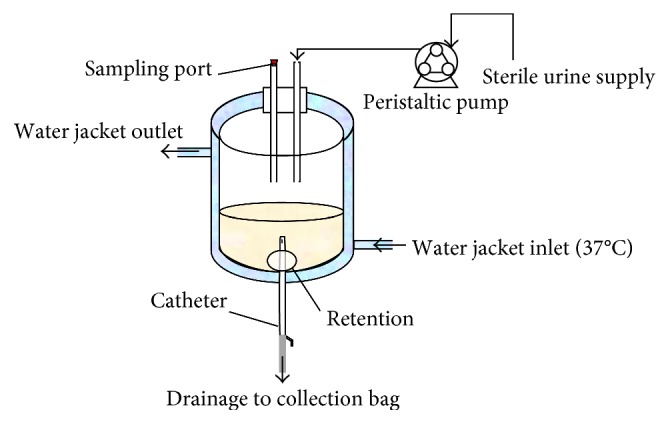
Schematic of an *in vitro* bladder model as first described by Stickler et al. [86].

Stickler et al. [86] developed a bladder model in 1999 comprising a glass vessel surrounded by a water jacket maintained at 37°C. The entire model is sterilised before an indwelling catheter is inserted into a glass outlet tube, and the retention balloon is inflated to keep the catheter in place before it is attached to a drainage bag (Figure 4) [86]. Sterile urine is then pumped into the model via a peristaltic pump and can then drain through the catheter into the attached drainage bag [86]. This model can and has been used to produce bacterial biofilms on outer and inner catheter surfaces and, in particular, crystalline biofilms where blockage of the catheter is the usual end point [86].

The Stickler et al. [86] model has been used and modified by several research studies to CAUTI developement, and test indwelling catheters entering the market containing or coated with novel antimicrobials [5, 10, 47, 48, 88].

#### 8.2.2. *In Vitro* Urinary Tract Model

**Figure 5:**
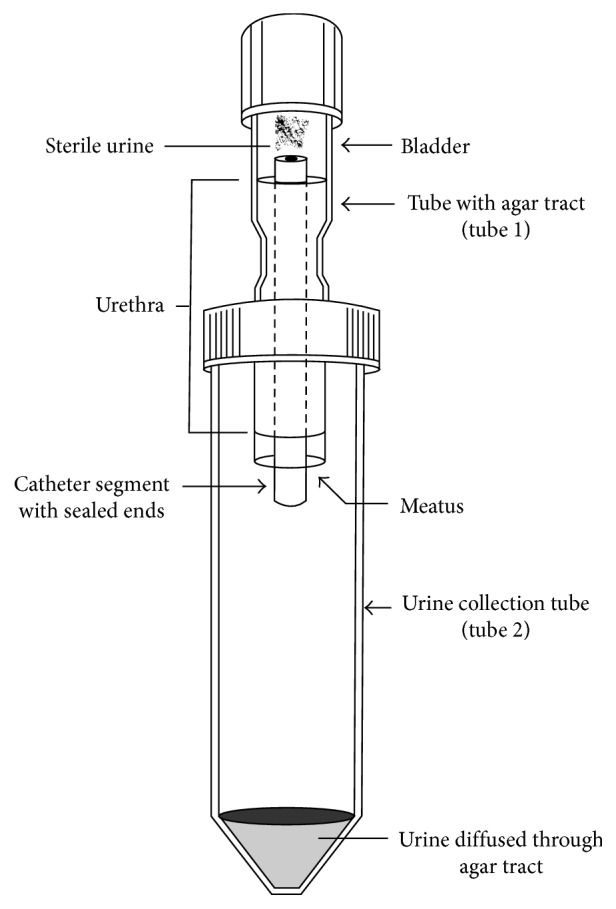
Gaonkar et al. [2] *in vitro* urinary tract model schematic.

Gaonkar et al. [2] developed a small scale model of the urinary tract to investigate the migration of pathogens along the surface of an indwelling catheter from the meatus to the bladder. The model consists of two tubes: tube 1 is an open narrower tube with a cap at one end and a rubber cork with a hole in it at the other end, and tube 2 is a larger vessel that is open at one end to connect to tube 1 and closed at the other end to collect urine; all parts are sterilised with ethylene dioxide [2]. The agar urethra portion is formed by placing a catheter segment aseptically into the top of tube 1 and secured by pushing through the hole in the rubber cork before molten agar is poured down the sides of the catheter, and once solidified, the rubber cork is removed. [2] Tube 1 is then secured in the top of tube 2 for testing (Figure 5).

The Gaonkar et al. [2] model methodology involves inoculating the “meatus” daily with bacteria, then filling the top of tube 1 with sterile urine daily. Samples are taken from the urine at the top of tube 1 periodically to assess if bacteria have migrated up the catheter and at what concentrations. This model is designed to test indwelling catheters over a number of days [2]. A modification of this model was used in a study by Williams and Stickler [89] to assess the migratory abilities of specific bacterial strains over a catheter surface. In their work, they left the bladder section of the model empty and replaced the trypticase soy agar used by Gaonkar et al. [2] with chromogenic UTI agar facilitating the visualisation of bacterial movement [89].

#### 8.2.3. *In Vitro* CAUTI Model

**Figure 6:**
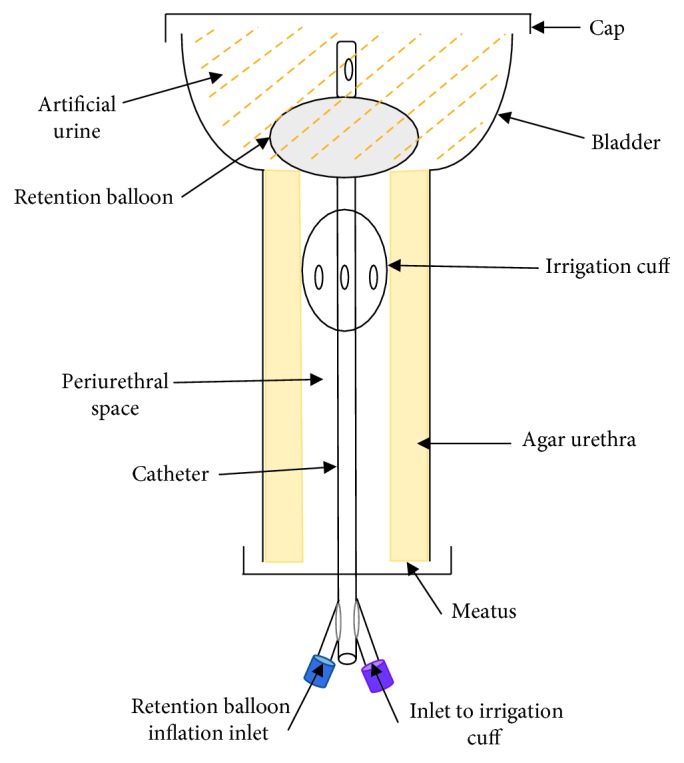
Illustration of the *in vitro* CAUTI model as described by Rosenblatt et al. [87].

Recently, the Gaonkar et al. [2] model has been adapted by Rosenblatt et al. [90]. Communicated on a poster presented in October 2017 at the IDweek conference in San Diego, California, Rosenblatt et al. [87] revealed their modified version of the urinary tract model or CAUTI model (Figure 6). This model consists of only one tube with an upper bulbous end to allow inflation of the retention cuff and a cap at the other end. The agar channel is wider than seen in the Gaonkar et al. [2] model, which allows to room the second catheter cuff or “proximal irrigation cuff,” which they modified the model to specifically test [58, 77]. The Rosenblatt et al. [87, 90] model methodology is similar to that of Gaonkar et al. [2], with the “meatus” being inoculated and the periurethral space then rinsed via the proximal irrigation cuff. The model was incubated to promote any growth, after which the catheter was removed, segmented, and the bioburden of the catheter assessed. Like the Gaonkar et al. [2] model, the Rosenblatt et al. [87] model is designed specifically for testing indwelling catheters, in particular, those with an irrigation cuff.

#### 8.2.4. Meatus Model

**Figure 7:**
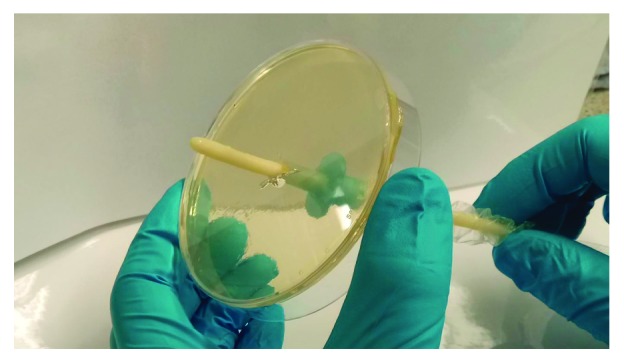
Laboratory reproduction of the Holland and Fish [39] *in vitro* meatus model. Catheter being inserted though the agar and through the bottom of the Petri plate.

A monograph study by Holland and Fish [39] developed a simple meatus model for the testing of intermittent catheters, specifically those with an introducer tip. The model consisted of an agar plate with one or more boreholes through both the agar and the plastic Petri plate [39]. The top surface of the agar acts as the outer tissue surrounding the urethra now as the meatus. The entire agar surface is inoculated with bacteria and a catheter is then passed through the agar and the boreholes. The portion of the catheter that has passed through the agar is cut off aseptically, and the bacteria is recovered from the surface of the catheter by sonication and bacterial enumeration is then carried out (Figure 7) [39].

## 9. Conclusion and Recommendations

With the historic and ongoing problems associated with catheter use, research and innovation is vital if CAUITs are to be controlled or eliminated [5, 6, 75]. ID development has stagnated since the development of the Foley catheter. Research has focused primarily on antimicrobials and new materials while there are still fundamental flaws with the basic design of the original Foley catheter. This contributes to CAUTI development by damaging the uroepithelial lining of the bladder, disrupting the bladder's immune defences, and allowing stagnation of urine with incomplete drainage of the bladder, thus not allowing full flushing of pathogens [91, 92, 93]. ICs on the other hand are often recommended to catheterised patients as a safer alternative to IDs, but they also have remained largely unchanged is modern medical history with a few noted exceptions [69]. Medical device research can be a costly and time-consuming venture that does not appeal to manufacturers; *in vitro* tests could bridge this gap [76, 94]. Although *in vitro* tests do not truly represent the anatomy and complex environment *in vivo,* they do offer many advantages including high throughput, relative low cost, low/no intersample (patient) variability, and no ethical concerns associated with *in vivo* tests [82]. Flexibility and timeframes for testing are also an important advantage, and developing novel *in vitro tests* or models that can be quickly adapted to suit the product being tested are of great interest [82].

The *in vitro* models discussed are examples of innovative yet convenient and simplistic *in vitro* tests that incorporate the basic structure of the urinary tract [28, 44–46]. They allow “proof of concept” testing before resources are wasted on more intensive and invasive *in vivo* or clinical testing [31]. During the research for this review, it became evident that with the exception of the model described by Holland and Fish [39], there are no specific *in vitro* urethral models that can be used to test intermittent catheters. The intermittent catheter design is focused on preventing bacteria entering the urinary tract or coming into contact with the catheter, and there is no standardised *in vitro* model to aid in the design of novel intermittent catheters or test the efficacy of manufacturer's CAUTI prevention claims [69].
